# Data analysis for COVID-19 deaths using a novel statistical model: Simulation and fuzzy application

**DOI:** 10.1371/journal.pone.0283618

**Published:** 2023-04-10

**Authors:** El-Sayed A. El-Sherpieny, Ehab M. Almetwally, Abdisalam Hassan Muse, Eslam Hussam

**Affiliations:** 1 Faculty of Graduate Studies for Statistical Research, Cairo University, Giza, Egypt; 2 Department of Statistics, Faculty of Business Administration, Delta University for Science and Technology, Gamasa, Egypt; 3 Faculty of Science and Humanities, School of Postgraduate Studies and Research (SPGSR), Amoud University, Borama, Somalia; 4 Department of Mathematics, Faculty of Science, Helwan University, Cairo, Egypt; University of Bradford, UNITED KINGDOM

## Abstract

This paper provides a novel model that is more relevant than the well-known conventional distributions, which stand for the two-parameter distribution of the lifetime modified Kies Topp–Leone (MKTL) model. Compared to the current distributions, the most recent one gives an unusually varied collection of probability functions. The density and hazard rate functions exhibit features, demonstrating that the model is flexible to several kinds of data. Multiple statistical characteristics have been obtained. To estimate the parameters of the MKTL model, we employed various estimation techniques, including maximum likelihood estimators (MLEs) and the Bayesian estimation approach. We compared the traditional reliability function model to the fuzzy reliability function model within the reliability analysis framework. A complete Monte Carlo simulation analysis is conducted to determine the precision of these estimators. The suggested model outperforms competing models in real-world applications and may be chosen as an enhanced model for building a statistical model for the COVID-19 data and other data sets with similar features.

## 1 Introduction

The modeling of lifespan distributions has received considerable attention over many years and decades. Because of the pertinence of modeling events and pandemics, there has been consistent growth throughout the course of time in the interest in modeling for lifespan distributions.

Distribution theory researchers often model for data in two ways: adding a new parameter to the distribution of interest to make it more flexible or constructing a new distribution family. This is done to provide the maximum flexibility possible given the distribution.

Modeling is fascinating in various fields, including manufacturing, engineering, reliability, and health research. See (Anake et al. [1]) for more reading.

This article provides the statistical modeling of the COVID-19 death certificates in the Kingdom of Saudi Arabia. If you are interested in reading more about this point of research, you may want to explore the following: Kumar [2], Khakharia et al. [3], Wang [4], Lalmuanawma et al. [5] and Bullock et al. [6].

Hassan et al. [7] was the first one to introduce the mathematical formulas for the cumulative distribution function (CDF) and probability density function (PDF) of the Topp-Leone distribution (TL) distribution with the shape parameter *β* > 0, as shown in the upcoming equation:

$$\begin{array}{c} G\left(x;\beta \right)=x^{\beta }{\left(2-x\right)}^{\beta };\;\;\;\;\;x>0,\beta >0, \end{array}$$ (1)

and,

$$\begin{array}{c} g\left(x;\beta \right)=2\beta x^{\beta -1}\left(1-x\right){\left(2-x\right)}^{\beta -1};\;\;\;\;\;x,\beta >0 \end{array}$$ (2)

Kumar and Dharmaja [8] investigated the features of the exponents Kies distribution. See Dey et al. [9] for more reading about modified Kies distribution. Al-Babtain et al. [10] introduced a novel family of distributions based on the modified Kies (MK) distribution family. If *G*(*z*;*δ*) is considered as the CDF of the baseline, which depends on a parameter vector called *δ* so we can express and describe the CDF of the MK family with the following equation:

$$\begin{array}{c} F\left(x;\Theta \right)=1-e^{-{\left[\frac{G(x;\Delta )}{1-G(x;\Delta )}\right]}^{\theta }},x>0,\theta >0, \end{array}$$ (3)

where Θ is vector contains the parameter (*θ*, Δ) and Δ is parameter vector of *G*(*x*).

We can express the PDF of Eq (3) like below

$$\begin{array}{c} f\left(x;\Theta \right)=\theta \frac{g\left(x;\Delta \right)G{\left(x;\Delta \right)}^{\theta -1}}{{\left[1-G(x;\Delta )\right]}^{\theta +1}}e^{-{\left[\frac{G(x;\Delta )}{1-G(x;\delta )}\right]}^{\theta }},x>0,\theta >0, \end{array}$$ (4)

The two-parameter modified Kies-Topp-Leone (MKTL) distribution is derived in this article. The MKTL distribution has several beneficial features. Because it may be either negatively or positively skew or even symmetrical, the proposed MKTL distribution has a very versatile PDF, enabling more versatility. It can potentially behave as either a decrease, increase, bathtub, or reverse-J risk rate. An additional advantage of the distribution that has been claimed is that it has a precise closed-form CDF and is very simple to modify.

In light of these merits, the distribution is a great choice for use in various fields, such as the evaluation of biological organisms, durability, and financial statistics, amongst other distributions. This article discussed one real-data application and concluded, based on the modeling of the findings, that the new distribution is an ideal rival to many common and standard distributions with the same number of scale and shape parameters. This was determined by comparing the results of the new distribution to those of the common and standard distributions as an example of this is the Type II Power Topp-Leone inverse exponential, see (Bantan et al. [11]), and others like [12]), [13]), [14]), and modified Kies exponential distributions see (Al-Babtain et al. [10]).

In our further research, we want to develop a novel version of the bivariate modified Kies inverted Topp-Leone model, which will be based on copula see for more information [15–17].

The remainder of this study is organized as follows: In Section 2, The MKTL distribution is constructed. In Section 3, the MKTL distribution and some of its mathematical properties are the subject of this discussion. In Section 4, for the MKTL distribution, we obtain a technique for estimating its parameters. In Section 5, successfully attained Fuzzy Reliability. In Section 6, We acquire MKTL distribution simulated results. In Section 7, data analysis for real-world data is presented. The paper is summed up, and its conclusion is presented in Section 8.

## 2 MKTL distribution

In the next part, we will review the mathematical equations underpinning the suggested distribution. By using Eq (1) and substituting in Eq (3) the CDF for the MKTL distribution is defined:

$$\begin{array}{c} F\left(x;\Theta \right)=1-exp\{-\frac{x^{\beta \theta }{\left(2-x\right)}^{\beta \theta }}{{\left[1-x^{\beta }{\left(2-x\right)}^{\beta }\right]}^{\theta }}\},x>0,\theta ,\beta >0, \end{array}$$ (5)

and the PDF that corresponds to it may be accessed here

$$\begin{array}{c} f\left(x;\Theta \right)=2\beta \theta \frac{x^{\beta \theta -1}\left(1-x\right){\left(2-x\right)}^{\beta \theta -1}}{{\left[1-x^{\beta }{\left(2-x\right)}^{\beta }\right]}^{\theta +1}}exp\{-\frac{x^{\beta \theta }{\left(2-x\right)}^{\beta \theta }}{{\left[1-x^{\beta }{\left(2-x\right)}^{\beta }\right]}^{\theta }}\},x>0,\theta ,\beta >0. \end{array}$$ (6)

such that Θ represents the parameter vector for the parameters (*θ*, *β*).

The survival function of the MKTL distribution is shown as

$$\begin{array}{c} S\left(x;\Theta \right)=exp\{-\frac{x^{\beta \theta }{\left(2-x\right)}^{\beta \theta }}{{\left[1-x^{\beta }{\left(2-x\right)}^{\beta }\right]}^{\theta }}\} \end{array}$$ (7)

The hazard rate function (hr) of the MKTL distribution may be represented as

$$\begin{array}{c} hr\left(z;\Theta \right)=2\beta \theta \frac{x^{\beta \theta -1}\left(1-x\right){\left(2-x\right)}^{\beta \theta -1}}{{\left[1-x^{\beta }{\left(2-x\right)}^{\beta }\right]}^{\theta +1}}. \end{array}$$ (8)

**Fig 1:**
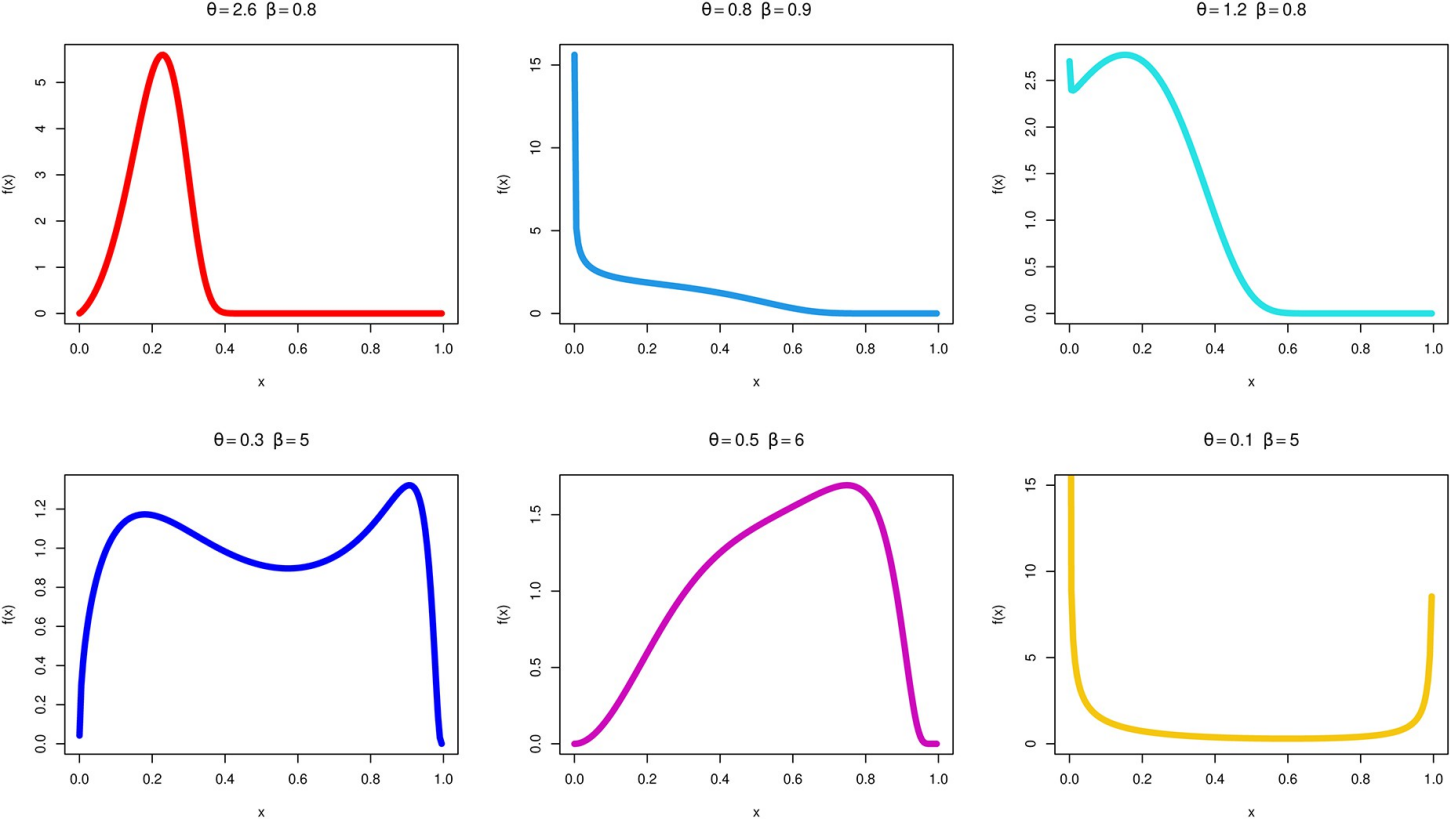
PDF of the MKTL distribution for certain parameter values.

**Fig 2:**
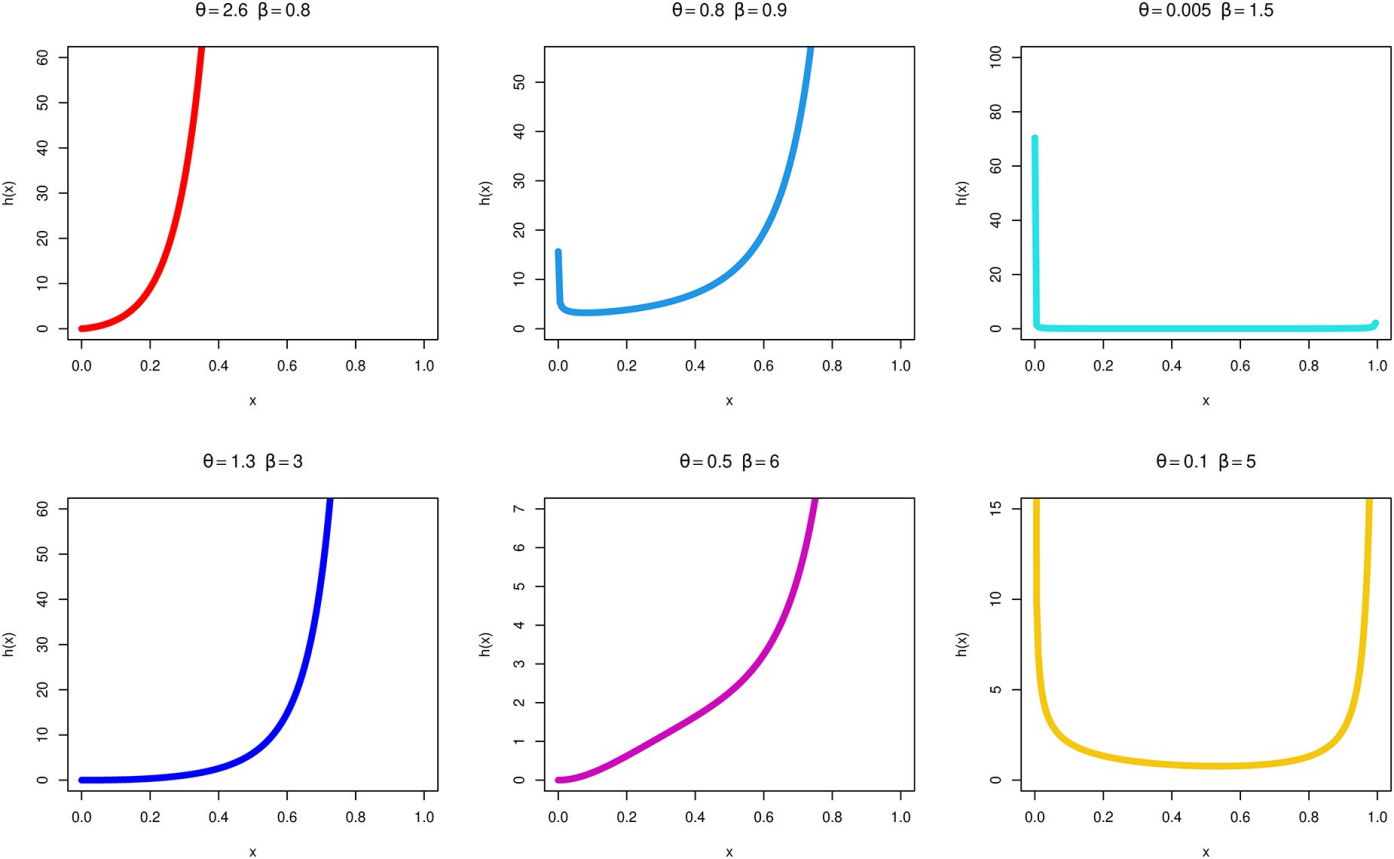
hr of the MKTL distribution for certain parameter values.

Figs 1 and 2 graph several plots of the MKTL distribution, using the values that have been provided for the parameters *θ* and *β*. It is clear from looking at the representations in Fig 2 that the MKTL distribution HR function may be scaled up or down or even modeled into the form of a bathtub. The TL distribution is a very poor model for data and phenomena that show the bathtub’s increasing and declining shapes and failure rates. That is one of the benefits of the MKTL distribution over the TL distribution. As a consequence of this, the MKTL distribution is in a stronger position than its competitor to analyze lifespan data.

## 3 Statistical properties of the proposed distribution of MKTL

### 3.1 A general expansion of the MKTL density

In this part, we give a linear representation of the MK family. Then we demonstrate how this representation may be used to produce a usable linear representation for the MKTL distribution. It is possible to offer the following as a depiction of the MK family’s mixture:

$$\begin{array}{c} f\left(x;\Theta \right)=\theta \sum_{j=0}^{\infty }\frac{{\left(-1\right)}^{j}}{j!}g\left(z;\delta \right)\frac{{\left[G(z;\delta )\right]}^{\theta (j+1)-1}}{{\left[1-G(z;\delta )\right]}^{\theta (j+1)+1}}. \end{array}$$ (9)

It is possible to rewrite the final equation of the MKTL distribution using the PDF and CDF values from the TL distribution.

f(x;Θ)=2βα(1-x)∑j,k=0∞(-1)j+kj!(α(j+1)+kk)xβ[α(j+1)+k]-1(2-x)β[α(j+1)+k]-1. (10)

As we can see the above equation number (10) represents the TL density using the parameter *β*[*α*(*j* + 1) + *k*].

### 3.2 Quantile for the MKTL distribution

The quantile function of the MKTL distribution, is derived by inverting Eq (5), like that we can see that *x* = *F*^−1^(*x*, Θ)(*U*) as follows:

$$\begin{array}{c} x=1-\sqrt{1-{\left(1+{\left[-ln(1-U)\right]}^{\frac{-1}{\theta }}\right)}^{\frac{-1}{\beta }}};\;0<U<1 \end{array}$$ (11)

using Eq (11) we can easily find the three quarterlies, also we can generate data using this equation.

### 3.3 Moments for the MKTL distribution

We can use equation number (10) to find the mathematical formula of the *r*_*th*_ moment like shown below for the variable *x*:

$$\begin{array}{cc} \overset{´}{\mu_{r}} & =E(x^{r})\\ & =2\beta \sum_{j,k,q=0}^{\infty }\eta_{j,k,q}\int_{0}^{1}x^{r}\left(1-x\right)x^{q}x^{\beta \left[\alpha (j+1)+k\right]-1}dx\\ & =2\beta \sum_{j,k,q=0}^{\infty }\eta_{j,k,q}\int_{0}^{1}\left(1-x\right)x^{r+q+\beta \left[\alpha (j+1)+k\right]-1}dx\\ & =2\beta \sum_{j,k,q=0}^{\infty }\eta_{j,k,q}Beta\left(r+q+\beta \left[\alpha \left(j+1\right)+k\right],2\right), \end{array}$$ (12)

where ηj,k,q=(β[α(j+1)+k]-1q)2β[α(j+1)+k]-q-1(-1)j+k+qj!(α(j+1)+kk).

## 4 Various approaches for estimation

We were successful in overcoming the challenges associated with estimating the MKTL distribution parameters by using a combination of Bayesian and non-Bayesian techniques throughout the estimation process. These methods are referred to by their individual names, such as maximum likelihood estimators (MLE) and Bayesian procedures based on squared error loss function (SELF).

### 4.1 Maximum likelihood estimators

Let it be assumed that the values *x*_1_, …, *x*_*n*_ are drawn at random from the MKTL distribution, which has the parameters *θ* and *β*. It can be shown that the likelihood function for the MKTL distribution is:

$$\begin{array}{c} L\left(\Theta \right)=2^{n}\beta^{n}\theta^{n}exp\{-\sum_{i=1}^{n}\frac{x_{i}^{\beta \theta }{\left(2-x_{i}\right)}^{\beta \theta }}{{\left[1-x_{i}^{\beta }{\left(2-x_{i}\right)}^{\beta }\right]}^{\theta }}\}\prod_{i=1}^{n}\frac{x_{i}^{\beta \theta -1}\left(1-x_{i}\right){\left(2-x_{i}\right)}^{\beta \theta -1}}{{\left[1-x_{i}^{\beta }{\left(2-x_{i}\right)}^{\beta }\right]}^{\theta +1}} \end{array}$$ (13)

The following formula may be used to get the log-likelihood function for the MKTL distribution:

$$\begin{array}{cc} l(\Theta )\propto & n\left[\text{ln}(\theta )+\text{ln}(\beta )\right]-\sum_{i=1}^{n}\frac{x_{i}^{\beta \theta }{\left(2-x_{i}\right)}^{\beta \theta }}{{\left[1-x_{i}^{\beta }{\left(2-x_{i}\right)}^{\beta }\right]}^{\theta }}+\left(\beta \theta -1\right)\sum_{i=1}^{n}\text{ln}\left(x_{i}\right)+\beta \theta -1\sum_{i=1}^{n}\text{ln}\left(2-x_{i}\right)\\ & -\left(\theta +1\right)\sum_{i=1}^{n}\text{ln}\left[1-x_{i}^{\beta }{\left(2-x_{i}\right)}^{\beta }\right] \end{array}$$ (14)

We will find the first derivatives for the above equations as find below:

$$\begin{array}{cc} \frac{\partial l(\Theta )}{\partial \theta }= & \frac{n}{\theta }-\sum_{i=1}^{n}\frac{x_{i}^{\beta \theta }{\left(2-x_{i}\right)}^{\beta \theta }\{\beta \left[\text{ln}(x)-\text{ln}(2-x)\right]-\text{ln}\left[1-x_{i}^{\beta }{\left(2-x_{i}\right)}^{\beta }\right]\}}{{\left[1-x_{i}^{\beta }{\left(2-x_{i}\right)}^{\beta }\right]}^{\theta }}+\beta \sum_{i=1}^{n}\text{ln}\left(x_{i}\right)\\ & +\beta \sum_{i=1}^{n}\text{ln}\left(2-x_{i}\right)-\sum_{i=1}^{n}\text{ln}\left[1-x_{i}^{\beta }{\left(2-x_{i}\right)}^{\beta }\right] \end{array}$$ (15)

and

$$\begin{array}{cc} \frac{\partial l(\Theta )}{\partial \beta }= & \frac{n}{\beta }-\sum_{i=1}^{n}\frac{x_{i}^{\beta \theta }{\left(2-x_{i}\right)}^{\beta \theta }\theta \left[\text{ln}(x)-\text{ln}(2-x)\right]}{{\left[1-x_{i}^{\beta }{\left(2-x_{i}\right)}^{\beta }\right]}^{\theta }}+\left(\theta +1\right)\sum_{i=1}^{n}\frac{x_{i}^{\beta }\text{ln}\left(x_{i}\right){\left(2-x_{i}\right)}^{\beta }\text{ln}\left(2-x_{i}\right)}{1-x_{i}^{\beta }{\left(2-x_{i}\right)}^{\beta }}\\ & +\sum_{i=1}^{n}\frac{x_{i}^{\beta \theta }{\left(2-x_{i}\right)}^{\beta \theta }\theta \left[\text{ln}(x)-\text{ln}(2-x)\right]{\left[1-x_{i}^{\beta }{\left(2-x_{i}\right)}^{\beta }\right]}^{\theta -1}}{{\left[1-x_{i}^{\beta }{\left(2-x_{i}\right)}^{\beta }\right]}^{2\theta }}+\theta \sum_{i=1}^{n}\text{ln}\left(2-x_{i}\right)+\theta \sum_{i=1}^{n}\text{ln}\left(x_{i}\right) \end{array}$$ (16)

Using the first derivative of Eq (14) with respect to *θ* and *β*, we can determine the MLE of these distribution parameters. Using the Newton-Raphson approach, which is implemented in the R program, the MLE may be optimized from the log-likelihood.

### 4.2 Bayesian estimation method

Bayesian estimation is one o the most important techniques for estimation. It considers the parameters as a random variable, by assuming that it has a prior distribution. We make the assumption that both *θ* and *β* have gamma priors. Also, The gamma priors for distribution parameters are respectively as follows:

$$\begin{array}{c} C_{1}\left(\theta \right)\propto \theta^{\varphi_{1}-1}e^{-D_{1}\theta },\;\;\;\;\theta >0,\;\varphi_{1},D_{1}>0, \end{array}$$ (17)

and

$$\begin{array}{c} C_{2}\left(\beta \right)\propto \beta^{\varphi_{2}-1}e^{-D_{2}\beta },\;\;\;\;\beta >0,\;\varphi_{2},D_{2}>0, \end{array}$$ (18)

Given that it is commonly known that the two priors are not dependent on one another, the joint prior of *θ* and *β* may be derived as follows:

$$\begin{array}{c} C\left(\theta ,\beta \right)\propto \theta^{\varphi_{1}-1}\beta^{\varphi_{2}-1}\;\text{exp}\left\{-\left(\theta D_{1}+\beta D_{2}\right)\right\},\;\;\;\;\theta ,\beta >0. \end{array}$$ (19)

We may utilize the estimate and variance-covariance matrix of the MLE approach in order to obtain adequate and superior values for the hyper-parameters of the independent joint prior. The estimated hyper-parameters may be stated as follows after the mean and variance of the gamma priors have been equated.

$$\begin{array}{c} \varphi_{j}=\frac{{\left[\frac{1}{B}\sum_{l=1}^{B}\hat{\Omega_{j}^{i}}\right]}^{2}}{\frac{1}{B-1}\sum_{l=1}^{B}{\left[\hat{\Omega_{j}^{i}}-\frac{1}{B}\sum_{l=1}^{B}\hat{\Omega_{j}^{i}}\right]}^{2}};\;j=1,2, \end{array}$$

$$\begin{array}{c} D_{j}=\frac{\frac{1}{B}\sum_{l=1}^{B}\hat{\Omega_{j}^{i}}}{\frac{1}{B-1}\sum_{l=1}^{B}{\left[\hat{\Omega_{j}^{i}}-\frac{1}{B}\sum_{l=1}^{B}\hat{\Omega_{j}^{i}}\right]}^{2}};\;j=1,2, \end{array}$$

wherein *B* is the number of times the repetition is performed.

The posterior distribution $C^{*}\left(\theta ,\beta \right)$ is constructed in the following way

$$\begin{array}{c} \begin{array}{c} C^{*}\left(\theta ,\beta \right)\propto \theta^{n+\varphi_{1}-1}\beta^{n+\varphi_{2}-1}exp\{-\left(\theta D_{1}+\beta D_{2}\right)-\sum_{i=1}^{n}\frac{x_{i}^{\beta \theta }{\left(2-x_{i}\right)}^{\beta \theta }}{{\left[1-x_{i}^{\beta }{\left(2-x_{i}\right)}^{\beta }\right]}^{\theta }}\}\prod_{i=1}^{n}\frac{x_{i}^{\beta \theta -1}\left(1-x_{i}\right){\left(2-x_{i}\right)}^{\beta \theta -1}}{{\left[1-x_{i}^{\beta }{\left(2-x_{i}\right)}^{\beta }\right]}^{\theta +1}}, \end{array} \end{array}$$ (20)

It is well known that *u*(Θ) = *u*(*θ*, *β*). The squared error loss function is used for Bayesian estimates of distribution parameters as below:

$$\begin{array}{c} {\tilde{u}}_{SE}\left(\Theta \right)=E\left(u\left(\Theta \right)\right), \end{array}$$ (21)

It is obvious that SELF estimations of *u*(*θ*, *β*) in (21), are very hard and look impossible to calculate. Since calculating numerous integrals analytically or even mathematically by hand is notoriously difficult, we utilized Mathematica 12 to employ an approximation approach that has shown to be highly helpful in solving these types of integration: the SELF estimator.

Therefore, the Markov chain Monte Carlo (MCMC) approach was used to approximately determine integrals in this setting. The Metropolis-Hastings (MH) algorithm, also known as the random walk algorithm, is a key part of MCMC. In a manner analogous to acceptance-rejection sampling, the MH algorithm takes into account the possibility that a candidate value might be formed from a proposal distribution at each stage of the process. Conditional posterior densities of MKTL are used to produce random samples, which are then analyzed using the MH method as follows:

$$\begin{array}{c} \begin{array}{c} C^{*}\left(\theta |\beta ,x\right)\propto \theta^{n+\varphi_{1}-1}exp\{-\theta D_{1}-\sum_{i=1}^{n}\frac{x_{i}^{\beta \theta }{\left(2-x_{i}\right)}^{\beta \theta }}{{\left[1-x_{i}^{\beta }{\left(2-x_{i}\right)}^{\beta }\right]}^{\theta }}\}\prod_{i=1}^{n}\frac{x_{i}^{\beta \theta -1}{\left(2-x_{i}\right)}^{\beta \theta -1}}{{\left[1-x_{i}^{\beta }{\left(2-x_{i}\right)}^{\beta }\right]}^{\theta +1}}, \end{array} \end{array}$$ (22)

and

$$\begin{array}{c} \begin{array}{c} C^{*}\left(\beta |\theta ,x\right)\propto \beta^{n+\varphi_{2}-1}exp\{-\beta D_{2}-\sum_{i=1}^{n}\frac{x_{i}^{\beta \theta }{\left(2-x_{i}\right)}^{\beta \theta }}{{\left[1-x_{i}^{\beta }{\left(2-x_{i}\right)}^{\beta }\right]}^{\theta }}\}\prod_{i=1}^{n}\frac{x_{i}^{\beta \theta -1}{\left(2-x_{i}\right)}^{\beta \theta -1}}{{\left[1-x_{i}^{\beta }{\left(2-x_{i}\right)}^{\beta }\right]}^{\theta +1}}, \end{array} \end{array}$$ (23)

For further detail, read Chen and Shao [18]. A confidence interval (CI) that is used by Bayesian estimators is referred to as the credible interval or, alternatively, as the highest posterior density (HPD) interval. They took advantage of a method that has seen a lot of usages elsewhere to generate HPD estimates for distribution characteristics that were unknown to them. It is recommended that estimates be generated using samples drawn using the MH algorithm that has been presented; for further details on the proposed approach, see Chen and Shao [18].

## 5 Fuzzy history

The ideas of durability and HR function could be viewed as probabilities that define a lifetime; nevertheless, the scope of HR functions has recently expanded as a result of the incorporation of hazy components into these functions. In conventional models of system dependability, the mortality rate probability of the parts involved is represented as actual numerical values. This is done in order to better understand the system’s behavior. However, this precision of system lifetimes does not hold true in the actual world because the values of system parameters collected by experimentation or guesswork are all susceptible to some degree of error.

It is feasible to utilize the fuzzy set theory in order to explain the real world in a way that is both realistic and practical. This is made possible by the fact that it is possible to apply the theory. As a direct result of this, putting into practice the idea of fuzziness will ultimately end up being more acceptable. Because of this, the idea of fuzziness has to be taken into consideration whether one is analyzing the behavior of a system or talking about the dependability of a system.

In order to generate fuzzy parameters, first, the membership functions are applied to characterize the level of fuzziness associated with the lifespan information or system parameters, and then the results of that characterization are used in the process of producing the fuzzy parameters. This is done so that the production of fuzzy parameters will be easier to do. One day, the fuzzy set theory may be used to explain the actual world in a manner that is not only believable but also helpful. This will be the case when it is applied. This is only one example of the many different ways in which the idea may be used.

### 5.1 Concepts and relationships in the science of fuzzy sets

In addition to the reliability of the probability distribution, the kind of data utilized to estimate the parameters is an incredibly crucial component in influencing the correctness of the findings that we reached. As a consequence of this, the data type must be specified. One of these types of data is known as fuzzy data, and it is one of the more recent and important advances in the field of statistics. This is due to the fact that many events in the real world do not have clear bounds. So suppose that *T* is a continuous random variable that represents a system’s failure time (component). Sabry et al. [19] introduced the inference of fuzzy reliability model for inverse Rayleigh distribution. Tolba et al. [20] discussed fuzzy statistical inference for stress-strength reliability using inverse Lomax lifetime distribution. Mohamed et al. [21] obtained fuzzy inference of reliability analysis for Type II Half logistic Weibull distribution. Meriem et al. [22] derived statistical inference, and fuzzy reliability for power xlindley distribution. We can easily calculate the fuzzy dependability using the fuzzy probability formula, see Chen and Pham [23]

$$\begin{array}{c} \tilde{R}\left(t\right)=P(T>t)=\int_{t}^{\infty }\mu \left(x\right)\;f\left(x\right)dx,0\leq t\leq x<\infty , \end{array}$$ (24)

where *μ*(*x*) is a membership function, For more reading see [24]

Then suppose that *μ*(*x*) is

$$\begin{array}{c} \mu \left(x\right)=\{\begin{array}{cc} 0 & ,x\leq t_{1}\\ \frac{x-t_{1}}{t_{2}-t_{1}} & ,t_{1}<x<t_{2,}t_{1}\geq 0\\ 1 & ,x\geq t_{2} \end{array} \end{array}$$ (25)

The lifespan *x*(*γ*) for *μ*(*x*), may be computed using the computing technique of the fuzzy numbers function and correlates to a given value of *γ* − *Cut*, *γ* ∈ [0, 1], , as follows: Chen and Pham [23],

$\mu \left(x\right)=\gamma \to \;\frac{x-t_{1}}{t_{2}-t_{1}}=\gamma ,\;$ then

$$\begin{array}{c} \left\{ \begin{array}{cc} x(\gamma )\leq t & ,\gamma =0\\ x\left(\gamma \right)=t_{1}+\gamma \left(t_{2}-t_{1}\right) & ,0<\gamma <1\\ x\left(\gamma \right)\geq t_{2} & ,\gamma =1 \end{array}\right. \end{array}$$ (26)

As a result, the fuzzy reliability values may be determined for all *γ* values, We investigate the fuzzy reliability of MKTL distribution based on the fuzzy reliability definition as follows equations:

If *γ* = 0

$$\begin{array}{c} \tilde{R}{\left(t\right)}_{\gamma =0}=\int_{t_{1}}^{t_{1}}f\left(x\right)\;dx=0. \end{array}$$ (27)

If *γ* has value

$$\begin{array}{c} \begin{array}{cc} \tilde{R}{\left(t\right)}_{0<\gamma <1} & =\int_{t_{1}}^{x(\gamma )}f\left(x\right)\;dx\\ & =\int_{t_{1}}^{x(\gamma )}\mu \left(x\right)2\beta \theta \frac{x^{\beta \theta -1}\left(1-x\right){\left(2-x\right)}^{\beta \theta -1}}{{\left[1-x^{\beta }{\left(2-x\right)}^{\beta }\right]}^{\theta +1}}exp\{-\frac{x^{\beta \theta }{\left(2-x\right)}^{\beta \theta }}{{\left[1-x^{\beta }{\left(2-x\right)}^{\beta }\right]}^{\theta }}\}dx, \end{array} \end{array}$$ (28)

where *x*(*γ*) = *t*_1_ + *γ*(*t*_2_ − *t*_1_), and if *γ* = 1

$$\begin{array}{c} \begin{array}{cc} \tilde{R}{\left(t\right)}_{\gamma =1} & =\int_{t_{1}}^{t_{2}}f\left(x\right)\;dx\\ & =\int_{t_{1}}^{t_{2}}2\beta \theta \frac{x^{\beta \theta -1}\left(1-x\right){\left(2-x\right)}^{\beta \theta -1}}{{\left[1-x^{\beta }{\left(2-x\right)}^{\beta }\right]}^{\theta +1}}exp\{-\frac{x^{\beta \theta }{\left(2-x\right)}^{\beta \theta }}{{\left[1-x^{\beta }{\left(2-x\right)}^{\beta }\right]}^{\theta }}\}dx\\ & =exp\{-\frac{t_{1}^{\beta \theta }{\left(2-t_{1}\right)}^{\beta \theta }}{{\left[1-t_{1}^{\beta }{\left(2-t_{1}\right)}^{\beta }\right]}^{\theta }}\}-exp\{-\frac{{t_{2}}^{\beta \theta }{\left(2-t_{2}\right)}^{\beta \theta }}{{\left[1-{t_{2}}^{\beta }{\left(2-t_{2}\right)}^{\beta }\right]}^{\theta }}\}. \end{array} \end{array}$$ (29)

## 6 Simulation results

Here, we estimate the MKTL parameters using two different approaches and compare them to evaluate their relative performance in a simulated setting. For the parameters *θ* = (0.5, 1.5, 3) and *β*, we explore a range of sample sizes (*n* = 30, 50, 100). Here, we choose *N* = 5, 000 samples at random from the MKTL distribution. Average bias (Abias), mean squared error (MSE), lower and higher confidence intervals (CI), and coverage probability are calculated for each estimate (CP).

Abias, mean squared error, and confidence interval are used to compare the efficiency of various estimators; those with less MSE values are preferred. The R software package is used to get the simulated outcomes. Tables 1–4 exhibit the Abias, MSE, and CI for the MLE and Bayesian estimations. In addition, as the sample size grows, the average estimate produced by any estimating technique becomes closer and closer to the real parameter values.

**Table 1:** Simulation results when the value of *θ* = 3.

*θ* = 3				MLE					Bayesian			
*β*		*T*		Abias	MSE	Lower	Upper	CP	Abias	MSE	Lower	Upper
0.5	30		*θ*	0.0900	0.1486	2.3546	3.8253	95.40%	0.0690	0.0781	2.6698	3.6448
			*β*	-0.0012	6.12E-04	0.4503	0.5473	95.60%	0.0025	1.57E-04	0.4804	0.5270
		0.005	*R*_1_	-0.0004	2.97E-06	0.2629	1.7336	95.40%	0.0000	4.77E-07	2.6698	3.6448
		0.1	*R*_2_	-0.0012	0.0055	0.5807	0.6777	95.60%	0.0099	0.0014	0.4804	0.5270
		0.005	*hr*_1_	0.0762	0.1908	0.2050	1.2657	95.40%	-0.0195	0.0390	2.6698	3.6448
		0.1	*hr*_2_	0.3337	6.3102	11.9074	12.0043	95.60%	-0.1481	1.3878	0.4804	0.5270
	50		*θ*	0.0418	0.0638	2.5529	3.5306	94.80%	0.0400	0.0314	2.7314	3.3730
			*β*	-0.0006	2.98E-04	0.4655	0.5332	96.00%	0.0012	7.76E-05	0.4865	0.5191
		0.005	*R*_1_	-0.0002	1.10E-06	0.5096	1.4873	94.80%	0.0000	2.26E-07	2.7314	3.3730
		0.1	*R*_2_	-0.0010	0.0025	0.5956	0.6633	96.00%	0.0052	0.0007	0.4865	0.5191
		0.005	*hr*_1_	0.0333	0.0766	0.0014	0.9763	94.80%	-0.0171	0.0187	2.7314	3.3730
		0.1	*hr*_2_	0.1793	2.8176	11.7676	11.8352	96.00%	-0.0567	0.7094	0.4865	0.5191
	100		*θ*	0.0177	0.0243	2.7138	3.3216	95.20%	0.0157	0.0134	2.7892	3.2328
			*β*	-0.0005	1.53E-04	0.4752	0.5237	95.20%	0.0005	3.95E-05	0.4880	0.5128
		0.005	*R*_1_	-0.0001	3.38E-07	0.6947	1.3025	95.20%	0.0000	1.23E-07	2.7892	3.2328
		0.1	*R*_2_	-0.0013	0.0012	0.6049	0.6534	95.20%	0.0022	0.0003	0.4880	0.5128
		0.005	*hr*_1_	0.0153	0.0266	0.1656	0.7734	95.20%	-0.0051	0.0100	2.7892	3.2328
		0.1	*hr*_2_	0.1124	1.4306	11.7103	11.7587	95.20%	-0.0267	0.3614	0.4880	0.5128
2	30		*θ*	0.2081	0.3279	2.1615	4.2548	94.60%	0.0935	0.0936	2.5909	3.6929
			*β*	0.0012	0.0092	1.8132	2.1891	94.80%	0.0108	0.0022	1.9192	2.0942
		0.005	*R*_1_	-9.748E-11	2.875E-19	0.0467	2.0467	94.60%	-2.770E-12	4.623E-23	2.5909	3.6929
		0.1	*R*_2_	-4.182E-05	2.975E-08	0.8120	1.1878	94.80%	1.985E-07	1.623E-09	1.9192	2.0942
		0.005	*hr*_1_	8.404E-08	2.017E-13	1.0347	1.0467	94.60%	1.865E-09	5.027E-17	2.5909	3.6929
		0.1	*hr*_2_	1.641E-03	5.600E-05	0.1683	0.1927	94.80%	-9.904E-05	4.378E-06	1.9192	2.0942
	50		*θ*	0.0841	0.1223	2.4183	3.7500	94.40%	0.0366	3.412E-02	2.7275	3.4059
			*β*	-0.0030	0.0050	1.8585	2.1355	95.20%	0.0036	1.201E-03	1.9335	2.0669
		0.005	*R*_1_	-4.023E-11	1.335E-19	0.3342	1.6658	94.40%	-2.080E-12	1.575E-23	2.7275	3.4059
		0.1	*R*_2_	-2.757E-05	1.369E-08	0.8614	1.1384	95.20%	-1.814E-06	9.561E-10	1.9335	2.0669
		0.005	*hr*_1_	3.489E-08	8.993E-14	0.6466	0.6658	94.40%	1.164E-09	1.787E-17	2.7275	3.4059
		0.1	*hr*_2_	1.147E-03	2.754E-05	0.1343	0.1427	95.20%	4.536E-05	2.592E-06	1.9335	2.0669
	100		*θ*	0.0506	0.0535	2.6076	3.4936	95.40%	0.0251	1.391E-02	2.8145	3.2367
			*β*	-0.0003	0.0026	1.9001	2.0992	94.00%	0.0025	6.364E-04	1.9545	2.0537
		0.005	*R*_1_	-5.620E-12	4.091E-22	0.5570	1.4430	95.40%	-1.310E-12	2.461E-24	2.8145	3.2367
		0.1	*R*_2_	-9.154E-06	2.937E-09	0.9004	1.0995	94.00%	9.429E-07	4.001E-10	1.9545	2.0537
		0.005	*hr*_1_	4.699E-09	3.967E-16	-0.4430	0.4430	95.40%	3.441E-10	3.025E-18	2.8145	3.2367
		0.1	*hr*_2_	3.746E-04	7.205E-06	-0.0960	0.1030	94.00%	-7.386E-05	1.120E-06	1.9545	2.0537

**Table 2:** Simulation results when the value of *θ* = 1.5.

*θ* = 1.5				MLE					Bayesian			
*β*	n	T		Abias	MSE	Lower	Upper	CP	Abias	MSE	Lower	Upper
0.5	30		*θ*	0.1007	0.0684	1.1209	2.0805	97.14%	0.0467	0.0189	1.3884	1.8891
			*β*	0.0032	0.0011	0.2885	0.4178	94.29%	0.0075	0.0003	0.3263	0.3846
		0.005	*R*_1_	-0.0723	0.0017	0.4116	1.3713	97.14%	-0.0693	0.0006	1.3884	1.8891
		0.1	*R*_2_	-0.1171	0.0048	0.1752	0.3046	94.29%	-0.1060	0.0011	0.3263	0.3846
		0.005	*hr*_1_	6.2680	21.5913	14.9319	15.8915	97.14%	8.8745	6.6120	1.3884	1.8891
		0.1	*hr*_2_	2.7400	50.1917	18.0806	18.2100	94.29%	7.8865	5.1932	0.3263	0.3846
	50		*θ*	0.0450	0.0269	1.2323	1.8576	93.88%	0.0171	0.0085	1.3690	1.6855
			*β*	0.0009	0.0010	0.4391	0.5628	93.88%	0.0039	0.0002	0.4779	0.5316
		0.005	*R*_1_	0.0005	0.0002	0.6516	1.2768	93.88%	0.0015	0.0001	1.3690	1.6855
		0.1	*R*_2_	0.0038	0.0028	0.4490	0.5727	93.88%	0.0071	0.0007	0.4779	0.5316
		0.005	*hr*_1_	-0.1666	3.8083	5.6644	6.2897	93.88%	-0.2284	1.0299	1.3690	1.6855
		0.1	*hr*_2_	0.1570	1.2954	8.6505	8.7742	93.88%	-0.0604	0.2953	0.4779	0.5316
	100		*θ*	0.0346	0.0145	1.3071	1.7620	94.94%	0.0125	0.0039	1.4260	1.6589
			*β*	0.0052	0.0006	0.4564	0.5539	94.94%	0.0047	0.0002	0.4814	0.5347
		0.005	*R*_1_	0.0022	0.0001	0.7384	1.1933	94.94%	0.0018	0.0000	1.4260	1.6589
		0.1	*R*_2_	0.0100	0.0017	0.4682	0.5658	94.94%	0.0080	0.0005	0.4814	0.5347
		0.005	*hr*_1_	-0.3471	2.2505	5.5691	6.0240	94.94%	-0.2510	0.5958	1.4260	1.6589
		0.1	*hr*_2_	-0.0274	0.6766	8.4791	8.5766	94.94%	-0.0983	0.2067	0.4814	0.5347
1.5	30		*θ*	0.0937	0.0746	1.0906	2.0968	95.10%	0.0407	0.0204	1.3157	1.8126
			*β*	-0.0036	0.0206	1.2147	1.7780	94.40%	0.0193	0.0056	1.3845	1.6473
		0.15	*R*_1_	0.0006	0.0012	0.4291	1.4353	95.10%	0.0049	0.0003	1.3157	1.8126
		0.3	*R*_2_	0.0051	0.0061	0.3717	0.9350	94.40%	0.0130	0.0015	1.3845	1.6473
		0.15	*hr*_1_	-0.0324	0.1802	0.6082	1.6143	95.10%	-0.0704	0.0484	1.3157	1.8126
		0.3	*hr*_2_	0.1084	0.8512	4.0388	4.6021	94.40%	-0.0821	0.1551	1.3845	1.6473
	50		*θ*	0.0330	0.0302	1.1986	1.8673	95.10%	0.0137	0.0082	1.3518	1.6779
			*β*	0.0036	0.0111	1.2971	1.7100	96.00%	0.0132	0.0030	1.4119	1.6117
		0.15	*R*_1_	-0.0004	0.0007	0.5969	1.2655	95.10%	0.0023	0.0002	1.3518	1.6779
		0.3	*R*_2_	0.0029	0.0031	0.4446	0.8576	96.00%	0.0070	0.0008	1.4119	1.6117
		0.15	*hr*_1_	-0.0113	0.0945	0.7980	1.4667	95.10%	-0.0348	0.0234	1.3518	1.6779
		0.3	*hr*_2_	0.0195	0.3849	4.0251	4.4380	96.00%	-0.0597	0.0865	1.4119	1.6117
	100		*θ*	0.0218	0.0135	1.2977	1.7459	95.50%	0.0086	0.0035	1.4021	1.6171
			*β*	-0.0033	0.0050	1.3581	1.6352	96.00%	0.0039	0.0013	1.4460	1.5761
		0.15	*R*_1_	-0.0001	0.0003	0.7074	1.1556	95.50%	0.0010	0.0001	1.4021	1.6171
		0.3	*R*_2_	0.0002	0.0014	0.5098	0.7870	96.00%	0.0026	0.0003	1.4460	1.5761
		0.15	*hr*_1_	-0.0037	0.0445	0.9158	1.3640	95.50%	-0.0146	0.0109	1.4021	1.6171
		0.3	*hr*_2_	0.0384	0.1639	4.1119	4.3891	96.00%	-0.0147	0.0387	1.4460	1.5761

**Table 3:** Simulation results when the value of *θ* = 0.5.

*θ* = 0.5				MLE					Bayesian			
*β*	n	T		Abias	MSE	Lower	Upper	CP	Abias	MSE	Lower	Upper
1.5	30		*θ*	0.0400	0.0088	0.3714	0.7086	95.24%	0.0136	0.0019	0.4516	0.6060
			*β*	0.0308	0.1104	0.8746	2.1869	97.62%	0.0929	0.0484	1.3601	1.9055
		0.15	*R*_1_	0.0149	0.0050	0.5075	0.8447	95.24%	0.0224	0.0022	0.4516	0.6060
		0.3	*R*_2_	0.0069	0.0040	-0.1801	1.1322	97.62%	0.0186	0.0017	1.3601	1.9055
		0.15	*hr*_1_	0.0010	0.0774	2.0590	2.3962	95.24%	-0.0627	0.0222	0.4516	0.6060
		0.3	*hr*_2_	0.1487	0.1817	1.9435	3.2558	97.62%	-0.0082	0.0265	1.3601	1.9055
	50		*θ*	0.0272	0.0031	0.4305	0.6240	96.30%	0.0082	0.0007	0.4668	0.5594
			*β*	-0.0519	0.0477	1.0284	1.8677	94.44%	0.0095	0.0135	1.3645	1.7331
		0.15	*R*_1_	-0.0022	0.0021	0.5622	0.7557	96.30%	0.0050	0.0007	0.4668	0.5594
		0.3	*R*_2_	-0.0078	0.0017	0.0417	0.8810	94.44%	0.0029	0.0005	1.3645	1.7331
		0.15	*hr*_1_	0.0556	0.0322	2.1854	2.3790	96.30%	-0.0046	0.0059	0.4668	0.5594
		0.3	*hr*_2_	0.1470	0.0868	2.1783	3.0176	94.44%	0.0237	0.0113	1.3645	1.7331
	100		*θ*	0.0104	0.0013	0.4415	0.5794	96.15%	0.0043	0.0003	0.4733	0.5383
			*β*	0.0471	0.0283	1.2247	1.8696	96.15%	0.0496	0.0109	1.4213	1.7654
		0.15	*R*_1_	0.0121	0.0009	0.6043	0.7422	96.15%	0.0115	0.0004	0.4733	0.5383
		0.3	*R*_2_	0.0088	0.0008	0.1554	0.8004	96.15%	0.0096	0.0003	1.4213	1.7654
		0.15	*hr*_1_	-0.0197	0.0152	2.1380	2.2759	96.15%	-0.0303	0.0048	0.4733	0.5383
		0.3	*hr*_2_	0.0207	0.0373	2.1491	2.7941	96.15%	-0.0123	0.0076	1.4213	1.7654
3	30		*θ*	0.0374	0.0092	0.3645	0.7103	94.60%	0.0124	0.0023	0.4358	0.6036
			*β*	0.0038	0.4138	1.7417	4.2659	95.80%	0.1609	0.1783	2.6395	4.0750
		0.15	*R*_1_	0.0037	0.0028	0.6934	1.0392	94.60%	0.0138	0.0013	0.4358	0.6036
		0.3	*R*_2_	0.0074	0.0046	-0.5784	1.9458	95.80%	0.0193	0.0021	2.6395	4.0750
		0.15	*hr*_1_	-0.0559	0.1089	1.1587	1.5045	94.60%	-0.1012	0.0533	0.4358	0.6036
		0.3	*hr*_2_	0.0197	0.0639	0.6142	3.1384	95.80%	-0.0498	0.0151	2.6395	4.0750
	50		*θ*	0.0125	0.0033	0.4019	0.6231	95.80%	0.0027	0.0008	0.4513	0.5576
			*β*	0.0173	0.2153	2.1076	3.9270	94.40%	0.0887	0.0738	2.6933	3.6166
		0.15	*R*_1_	0.0006	0.0015	0.7526	0.9738	95.80%	0.0064	0.0006	0.4513	0.5576
		0.3	*R*_2_	0.0025	0.0023	-0.2308	1.5885	94.40%	0.0090	0.0009	2.6933	3.6166
		0.15	*hr*_1_	-0.0220	0.0526	1.2549	1.4761	95.80%	-0.0465	0.0213	0.4513	0.5576
		0.3	*hr*_2_	0.0056	0.0251	0.9525	2.7718	94.40%	-0.0264	0.0061	2.6933	3.6166
	100		*θ*	0.0047	0.0017	0.4254	0.5839	95.80%	0.0003	0.0004	0.4629	0.5419
			*β*	-0.0148	0.1093	2.3374	3.6331	94.80%	0.0353	0.0319	2.7196	3.3694
		0.15	*R*_1_	-0.0025	0.0008	0.7808	0.9394	95.80%	0.0020	0.0003	0.4629	0.5419
		0.3	*R*_2_	-0.0020	0.0012	0.0265	1.3222	94.80%	0.0031	0.0004	2.7196	3.3694
		0.15	*hr*_1_	0.0040	0.0261	1.3122	1.4708	95.80%	-0.0166	0.0097	0.4629	0.5419
		0.3	*hr*_2_	0.0096	0.0118	1.2184	2.5141	94.80%	-0.0109	0.0026	2.7196	3.3694

**Table 4:** Fuzzy reliability estimation by using MLE, and Bayesian method for MKTL distribution for *θ* = 0.5, 1.5 and 3.

L.Ms.F.					MLE					Bayesian			
(*t*_1_,*t*_2_)	*θ*	*β*	n	*γ*	Abias	MSE	Lower	Upper	CP	Abias	MSE	Lower	Upper
(0.1,0.75)	0.5	1.5	30	0.3	0.0074	0.0006	0.2274	0.3186	95.24%	0.0029	0.0001	0.2510	0.2908
				0.6	0.0217	0.0035	0.3875	0.6042	95.24%	0.0116	0.0010	0.4437	0.5460
				0.9	0.0234	0.0057	0.5333	0.8178	95.24%	0.0191	0.0021	0.6071	0.7554
			50	0.3	-0.0025	0.0001	0.2402	0.2858	100.00%	-0.0001	0.0001	0.2538	0.2788
				0.6	0.0031	0.0012	0.4074	0.5471	93.08%	0.0053	0.0005	0.4497	0.5335
				0.9	0.0123	0.0032	0.5510	0.7780	92.31%	0.0137	0.0014	0.6214	0.7552
			100	0.3	0.0033	0.0001	0.2486	0.2890	96.15%	0.0016	0.0000	0.2582	0.2774
				0.6	0.0087	0.0005	0.4395	0.5262	93.08%	0.0053	0.0002	0.4546	0.4964
				0.9	0.0119	0.0009	0.6101	0.7181	92.31%	0.0090	0.0003	0.6311	0.6790
		3	30	0.3	-0.0070	0.0010	0.1707	0.2906	95.60%	-0.0100	0.0004	0.1881	0.2503
				0.6	0.0025	0.0016	0.4074	0.5618	94.20%	-0.0056	0.0003	0.4425	0.5135
				0.9	0.0132	0.0032	0.6327	0.8494	93.80%	0.0038	0.0009	0.6782	0.7917
			50	0.3	-0.0034	0.0004	0.1965	0.2720	93.60%	-0.0046	0.0001	0.2119	0.2502
				0.6	-0.0003	0.0005	0.4364	0.5271	93.60%	-0.0034	0.0001	0.4567	0.4985
				0.9	0.0032	0.0014	0.6583	0.8037	94.80%	0.0000	0.0004	0.6931	0.7656
			100	0.3	-0.0006	0.0002	0.2123	0.2617	95.00%	-0.0018	0.0001	0.2208	0.2472
				0.6	0.0001	0.0003	0.4501	0.5142	94.80%	-0.0017	0.0001	0.4680	0.4951
				0.9	0.0004	0.0008	0.6736	0.7828	95.20%	-0.0007	0.0002	0.7003	0.7542
(0.05,0.3)	1.5	0.5	30	0.3	0.0035	0.0013	0.2652	0.4089	96.15%	-0.0010	0.0004	0.3058	0.3765
				0.6	0.0092	0.0030	0.4791	0.6937	96.15%	0.0037	0.0009	0.5287	0.6329
				0.9	0.0082	0.0037	0.5891	0.8259	94.87%	0.0074	0.0010	0.6448	0.7696
			50	0.3	0.0034	0.0006	0.2870	0.3868	95.92%	0.0002	0.0002	0.3103	0.3634
				0.6	0.0076	0.0021	0.4958	0.6738	93.88%	0.0037	0.0007	0.5374	0.6298
				0.9	0.0059	0.0025	0.6075	0.8028	95.92%	0.0055	0.0007	0.6616	0.7543
			100	0.3	0.0013	0.0003	0.2984	0.3714	97.47%	-0.0004	0.0001	0.3127	0.3537
				0.6	0.0070	0.0012	0.5182	0.6501	94.94%	0.0029	0.0003	0.5543	0.6222
				0.9	0.0090	0.0014	0.6354	0.7811	94.94%	0.0055	0.0004	0.6633	0.7371
		1.5	30	0.3	-0.0015	0.0003	0.0027	0.0728	96.50%	-0.0028	0.0001	0.0188	0.0523
				0.6	-0.0058	0.0017	0.0429	0.2038	96.00%	-0.0073	0.0005	0.0891	0.1639
				0.9	-0.0100	0.0039	0.1506	0.3919	97.00%	-0.0116	0.0010	0.2118	0.3224
			50	0.3	-0.0012	0.0002	0.0079	0.0681	96.50%	-0.0021	0.0001	0.0215	0.0496
				0.6	-0.0043	0.0012	0.0563	0.1932	95.50%	-0.0053	0.0003	0.0903	0.1537
				0.9	-0.0066	0.0027	0.1743	0.3751	94.50%	-0.0080	0.0007	0.2205	0.3118
			100	0.3	0.0006	0.0001	0.0198	0.0599	94.50%	-0.0003	0.0000	0.0286	0.0482
				0.6	0.0007	0.0005	0.0841	0.1755	93.00%	-0.0011	0.0001	0.1047	0.1498
				0.9	0.0007	0.0011	0.2156	0.3483	93.50%	-0.0019	0.0003	0.2443	0.3100
(0.05,0.3)	3	0.5	30	0.3	0.0112	0.0025	0.3882	0.5804	96.50%	0.0024	0.0006	0.4305	0.5218
				0.6	0.0049	0.0014	0.8072	0.9554	96.00%	0.0080	0.0006	0.8418	0.9330
				0.9	0.0000	0.0012	0.8426	0.9786	97.00%	0.0053	0.0003	0.8862	0.9522
			50	0.3	0.0033	0.0015	0.4012	0.5515	97.00%	-0.0006	0.0004	0.4367	0.5120
				0.6	-0.0025	0.0008	0.8194	0.9285	94.50%	0.0022	0.0003	0.8458	0.9153
				0.9	-0.0025	0.0006	0.8608	0.9555	94.50%	0.0022	0.0002	0.8896	0.9411
			100	0.3	0.0035	0.0008	0.4231	0.5301	93.50%	0.0006	0.0002	0.4465	0.4996
				0.6	0.0017	0.0003	0.8472	0.9090	95.00%	0.0020	0.0001	0.8583	0.9005
				0.9	0.0001	0.0002	0.8798	0.9417	93.00%	0.0013	0.0001	0.8924	0.9274
(0.20,0.5)		2	30	0.3	-0.0012	0.0003	-0.0027	0.0618	95.50%	-0.0022	0.0001	0.0138	0.0425
				0.6	-0.0087	0.0038	0.0710	0.3115	96.00%	-0.0094	0.0009	0.1265	0.2376
				0.9	-0.0022	0.0066	0.5422	0.8611	96.50%	-0.0066	0.0016	0.6151	0.7616
			50	0.3	-0.0005	0.0002	0.0050	0.0556	94.50%	-0.0012	0.0000	0.0195	0.0426
				0.6	-0.0046	0.0021	0.1064	0.2843	94.50%	-0.0050	0.0005	0.1599	0.2412
				0.9	0.0007	0.0025	0.6063	0.8027	94.50%	-0.0028	0.0007	0.6556	0.7496
			100	0.3	0.0001	0.0001	0.0140	0.0478	95.50%	-0.0003	0.0000	0.0228	0.0380
				0.6	-0.0015	0.0009	0.1404	0.2565	95.00%	-0.0017	0.0002	0.1714	0.2245
				0.9	-0.0023	0.0012	0.6343	0.7687	94.00%	-0.0030	0.0003	0.6625	0.7334

### 6.1 Observation and concluding remarks concerning the simulation and its results

1. When the sample size is increased, the mean values of the parameters move closer to their original values, and the mean squared error (MSE) falls.
2. The performance of all estimators is quite high. They all estimate very tiny MSE and mean values similar to the original parameters.
3. In terms of MSE values and parameter mean values, the differences between all estimators are relatively modest.

## 7 Real data analysis

This part aims to demonstrate the MKTL distribution’s applicability to one set of actual data. When compared to other competing models, MKTL distribution is examined, namely: Kumaraswamy, Beta, Gompertz Lomax (GL) (Oguntunde et al. [13]), Topp-Leaon generalized exponential (TLGE), Type II Power Topp-Leone inverse exponential (TIIPTLIE), modified Kies exponential (MKEx), and alpha power inverted TL (APITL).

**Fig 3:**
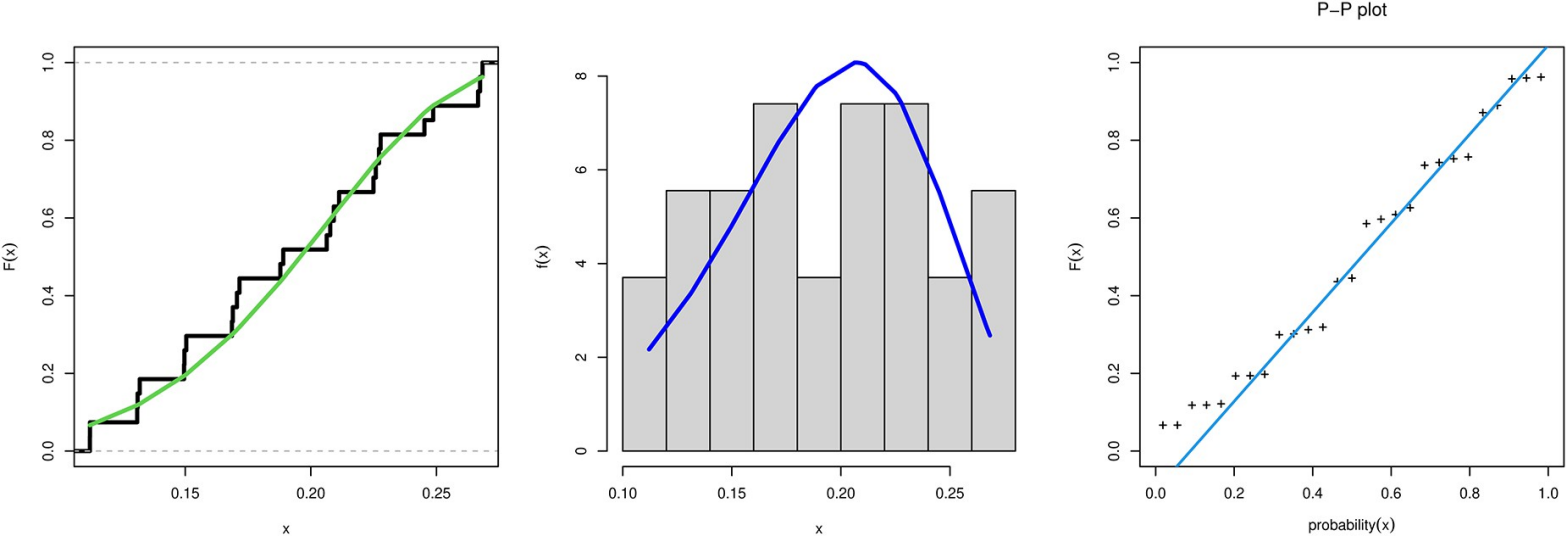
Cumulative function and empirical CDF, as well as the histogram and P-P plot for the MKTL distribution for the data under investigation.

Table 5 provide values of Cramér-von Mises (W), Anderson-Darling (A), and Kolmogorov- Smirnov (KS) statistic along with its P-value for all models fitted based on one real data sets. In addition, these tables contain the MLE and standard errors (SE) of the parameters for the considered models. In Table 5 results when the MKTL compared to all other models tried to fit the COVID-19, the MKTL distribution has the greatest P-value and the smallest distance of Kolmogorov-Smirnov(KS), W, and A value. Fig 3 show the fit empirical, histogram, and PP-plot for the MKTL distribution for the data under investigation.

**Table 5:** Values of estimates and all fitting results for Saudi Arabia mortality rate.

		estimates	SE	KS	P-Value	W	A
MKTL	*θ*	3.7670	0.5535	0.1254	0.7430	0.0539	0.3964
	*β*	0.7145	0.0277				
TIIPTLIE	*α*	51.3991	5.6616	0.1306	0.6979	0.0594	0.3979
	*θ*	0.4341	0.0745				
	*β*	1.1775	0.2698				
TLGE	*α*	0.3602	0.3504	0.1411	0.6059	0.0647	0.4305
	*θ*	18.0897	3.0623				
	*β*	72.5703	84.2444				
K	*α*	3.0944	0.3293	0.1335	0.6727	0.0597	0.3985
	*θ*	125.1654	61.5947				
beta	*α*	12.7883	3.4371	0.1272	0.7277	0.0572	0.3968
	*θ*	54.1398	14.7710				
GL	*α*	3.7768	3.2167	0.1299	0.7041	0.0629	0.4646
	*θ*	5.8215	5.4013				
	*β*	1.3181	1.2191				
	λ	0.0163	0.0196				
APITL	*α*	537.2229	582.5309	0.1274	0.7261	0.0637	0.4234
	*θ*	89.8798	10.6103				
MKEx	*α*	3.4889	0.5422	0.1312	0.6930	0.0542	0.3982
	*θ*	3.2790	0.1378				

The data represents a COVID-19 data belong to Saudi Arabia of 27 days, from 4 August 2021 to 30 August 2021 see the link https://covid19.who.int/.

**Fig 4:**
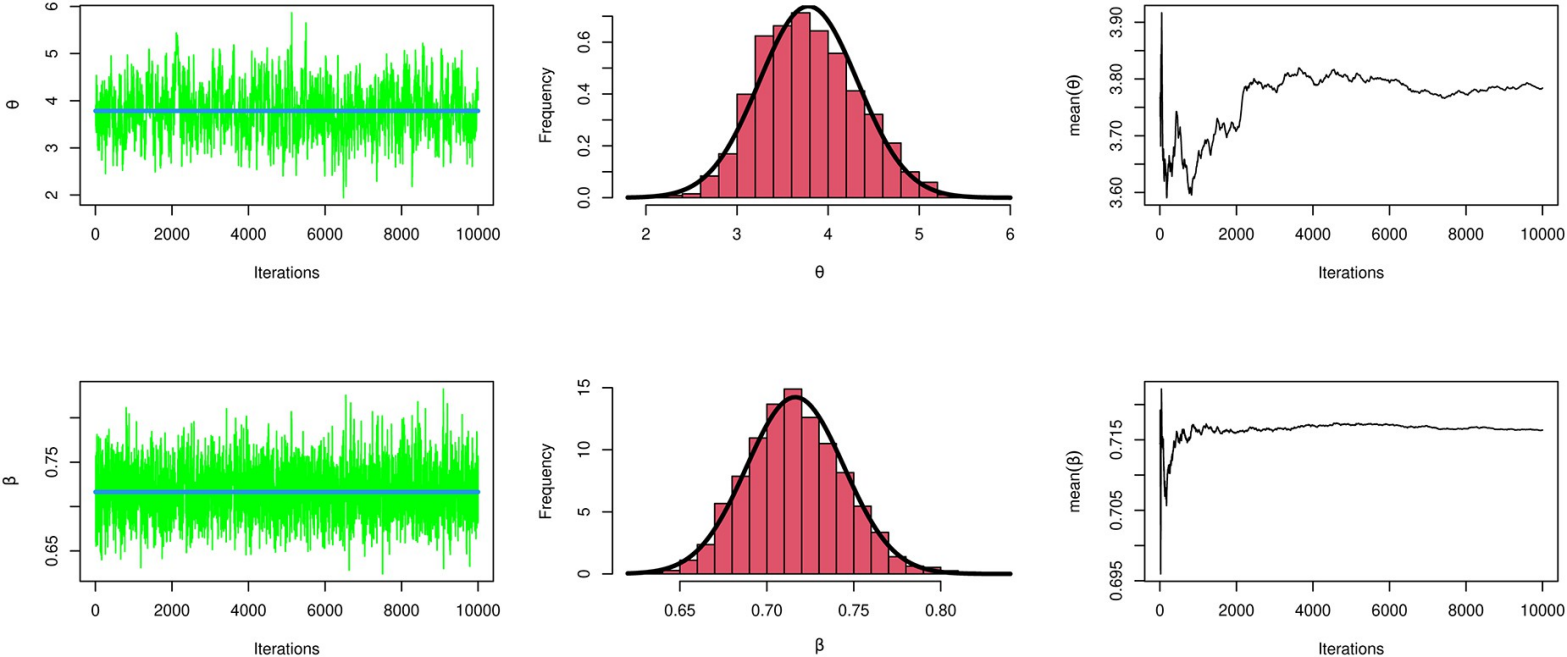
Convergence plots of MCMC for parameter estimates of MKTL distribution for the data under investigation.

These data formed of drought mortality rate. The data are as follows: 0.2113, 0.2683, 0.2487, 0.2674, 0.1716, 0.2666, 0.2091, 0.2278, 0.1706, 0.2271, 0.1890, 0.2077, 0.2452, 0.1319, 0.2259, 0.1504, 0.1879, 0.1689, 0.2063, 0.2249, 0.1686, 0.1310, 0.1497, 0.1309, 0.1495, 0.1121, and 0.1120. Results are tabulated in Table 5 and Fig 3. Fig 3 show Fuzzy Reliability when *t*_1_ = 0.12 and *t*_2_ = 0.25 with different value of *γ* cut plot and boxplot for the MKTL distribution for the data being analyzed. We note the Fuzzy Reliability increases as increases *γ* cut. Table 6 shows MLE and Bayesian for parameter estimates of MKTL distribution. Fig 4 display convergence charts of the Markov chain Monte Carlo method for parameter estimations of the MKTL distribution.

**Table 6:** MLE and Bayesian for parameter estimates of MKTL distribution for the data under investigation.

				CI		Fuzzy Reliability		
		estimates	SE	CIL1	CIU1	0.3	0.6	0.9
MLE	*θ*	3.7670	0.5535	2.6821	4.8520	0.1566	0.4311	0.7359
	*β*	0.7145	0.0277	0.6602	0.7687			
Bayesian	*θ*	3.7840	0.5401	2.7806	4.8380	0.1544	0.4278	0.7347
	*β*	0.7164	0.0268	0.6632	0.7701			

### 7.1 Conclusions and suggestions regarding the application

**Fig 5:**
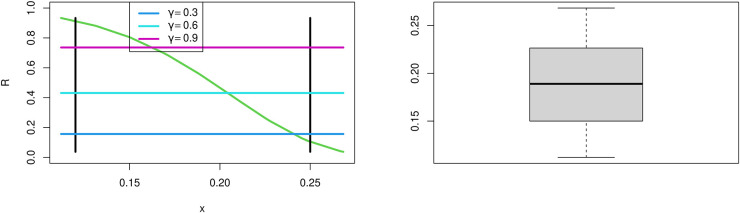
Fuzzy Reliability when *t*_1_ = 0.12 and *t*_2_ = 0.25 with different values of *γ* cut plot and boxplot for the MKTL distribution for the data under investigation.

1. From the data set, we can observe that MKTL yields the best P-value and the shortest W*, A*, and KS distances.
2. From Fig 3, We may conclude that MKTL was the best-fitting model for this dataset.
3. From Fig 5, we can deduce that Fuzzy Reliability when *t*_1_ = 0.12 and *t*_2_ = 0.25 with different value of *γ* cut plot, when *γ* increases then Fuzzy reliability increases. In the box plot for the MKTL distribution for the data under investigation, we note the data haven’t outliers.
4. Referring to Table 5, we can see that TIIPTLIE, TLGE, Kumaraswamy, beta, GL, APITL, and MKEx distribution provides good fitting for this data set, but the proposed model was the best.
5. Referring to Table 6, the Bayesian estimate technique is the most suitable estimation approach to use with this data.

## 8 Summary

This work introduces a novel two-parameter model we name the modified Kies Topp-Leone distribution (or MKTL distribution for short). When analyzing lifespan data, the MKTL distribution offers greater leeway than more standard distributions. The MKTL distribution’s hazard function, quantiles, and moments are shown along with its survival function and linear representation. A fuzzy reliability measure for the MKTL distribution has been derived. Our research shows that the Bayesian approach is superior to the MLE technique. We give an application for the COVID-19 mortality rate and show that the MKTL distribution is superior to other alternatives for fitting this data. To estimate the parameters of the MKTL distribution, MLE and Bayesian methods are used. To evaluate the model’s efficiency, we present estimating techniques, the findings of Fuzzy Reliability, and simulations. Compared to the TIIPTLIE, TLGE, Kumaraswamy, beta, GL, APITL, and MKEx distributions, The proposed model presented using real-world data shows a consistently superior fit.
